# Reduced misclosure of global sea-level budget with updated Tongji-Grace2018 solution

**DOI:** 10.1038/s41598-021-96880-w

**Published:** 2021-09-03

**Authors:** Fengwei Wang, Yunzhong Shen, Qiujie Chen, Yu Sun

**Affiliations:** 1 College of Surveying and Geo-Informatics, Tongji University, Shanghai, People’s Republic of China; 2 Key Lab of Spatial Data Mining and Information Sharing of Ministry of Education, Fuzhou University, Fuzhou, People’s Republic of China

**Keywords:** Physical oceanography, Geophysics

## Abstract

The global sea-level budget is studied using the Gravity Recovery and Climate Experiment (GRACE) solutions, Satellite Altimetry and Argo observations based on the updated budget equation. When the global ocean mass change is estimated with the updated Tongji-Grace2018 solution, the misclosure of the global sea-level budget can be reduced by 0.11–0.22 mm/year compared to four other recent solutions (i.e. CSR RL06, GFZ RL06, JPL RL06 and ITSG-Grace2018) over the period January 2005 to December 2016. When the same missing months as the GRACE solution are deleted from altimetry and Argo data, the misclosure will be reduced by 0.06 mm/year. Once retained the GRACE C_20_ term, the linear trends of Tongji-Grace2018 and ITSG-Grace2018 solutions are 2.60 ± 0.16 and 2.54 ± 0.16 mm/year, closer to 2.60 ± 0.14 mm/year from Altimetry–Argo than the three RL06 official solutions. Therefore, the Tongji-Grace2018 solution can reduce the misclosure between altimetry, Argo and GRACE data, regardless of whether the C_20_ term is replaced or not, since the low-degree spherical harmonic coefficients of the Tongji-Grace2018 solution can capture more ocean signals, which are confirmed by the statistical results of the time series of global mean ocean mass change derived from five GRACE solutions with the spherical harmonic coefficients truncated to different degrees and orders.

## Introduction

The global sea-level variations have been accurately measured by satellite altimetry since^1^ 1992, which mainly contain two components: mass-induced variation due to mass exchange among the oceans, land and atmosphere^2^ and steric variation due to the changes in seawater temperature and salinity, which can be directly measured by the Argo project (with a reasonable global spatial coverage after 2005)^3^. Therefore, by subtracting the steric variation from the total variation (Altimetry–Argo), one can derive the global ocean mass variation^4^. Since the launch of the twin- satellites of Gravity Recovery and Climate Experiment (GRACE) in 2002, the global ocean mass change can be directly estimated by using GRACE solutions^5^, which provided the possibility to investigate the misclosure of the global sea-level budget between the GRACE derived ocean mass and Altimetry–Argo one^6–8^.

Conventional sea-level budget equates the total Sea Surface Height (SSH) change to a sum of mass and steric sea level change^8^. The conventional sea-level budget equation is expressed as follows^9^,

1 $$ SL_{{{\text{total}}}} { = }SL_{{{\text{steric}}}} + SL_{{{\text{mass}}}} + \varepsilon $$

where $SL_{{{\text{steric}}}}$ refers to the contribution of ocean thermal expansion and salinity to sea-level change, and $SL_{{{\text{mass}}}}$ refers to the sea-level change due to the mass change in the oceans,$\varepsilon$ is the difference often described as the misclosure. Many previous studies tried to close the global sea-level budget, however, there exist significant differences among the results, mainly due to the larger uncertainty of early released GRACE solutions^4^. With the released GRACE RL05 and RL06 solutions, the misclosure of the global sea-level budget has been significantly reduced among GRACE, Argo and Altimetry data^4,10–12^, normally within the uncertainty (about 0.30 mm/year for trend) of the observations during the satellite era^13^. Since the ocean thermal expansion and salinity are only measured up to the upper 2000 m of the ocean by Argo floats and the ocean mass change will cause Ocean Bottom Deformation (OBD), Frederikse et al.^14^ and Vishwakarma et al.^9^ pointed out that OBD and deep-ocean steric sea-level change (> 2000 m) should be considered in the global sea-level budget equation as follows^9^,

2 $$ SL_{{{{total}}}} {{ - SL}}_{{{{OBD}}}} { = }SL_{{{\text{steric}}}} + SL_{{\text{deep steric}}} + SL_{{{\text{mass}}}} + \varepsilon $$

where *SL*_OBD_ denotes the effect of OBD, $SL_{{\text{deep steric}}}$ is the deep-ocean steric sea-level change. Since accurate quantification of the global sea-level budget is important for monitoring the global climate system and its long-term variability, one tries to reduce the misclosure and its uncertainty, especially for the estimation of the global ocean mass change^13^.

Compared to altimetry and Argo observations, accurate quantification of Global Mean Ocean Mass (GMOM) change by using GRACE solutions has been more challenging due to many affecting factors, for example, filtering method, signal leakage correction, Glacial Isostatic Adjustment (GIA) correction, geocenter motion correction, Earth oblateness correction, as well as pole tide correction, all have a substantial impact on ocean-mass estimates (e.g., Refs.^4,15–19^). Among these factors, Geocenter motion (Degree-1), C_20_ and GIA corrections have large impacts on the GMOM estimates^4,20,21^. According to Chen et al.^12^, the misclosure by using different post-processing strategies ranges from 0.18 to 0.51 mm/year relative to the results derived from Altimetry minus Argo for the period from January 2005 to December 2015.

Different processing strategies of GRACE solutions will bring certain differences in GMOM estimation, the four recently released GRACE solutions by the Center for Space Research (CSR), German Geoforschungszentrum (GFZ), Jet Propulsion Laboratory (JPL), Institute of Geodesy in University of Graz (i.e. CSR RL06, GFZ RL06, JPL RL06 and ITSG-Grace2018) were all developed with the dynamic approach, however, Tongji-Grace2018 was developed by using an optimized short-arc approach^22^. According to Meyer et al.^23^, the noise of the Tongji-Grace2018 solution, which is determined by variance component estimation, is relatively smaller in the combination of time-variable gravity fields including the other four GRACE solutions. Thus in this study, we will use the Tongji-Grace2018 solution to estimate the GMOM change and analyze the misclosure of the global sea-level budget (Eq. (2)) relative to the other four recently released solutions using the same post-processing strategies like that in Refs.^4^ and^12^. The rest of this paper is organized as follows: “Data and processing strategies” briefly introduced the Data sources and methods of this study. Results and analysis are presented in Sect. “Results and analysis” and finally, the concluding remarks are shown in “Conclusions” section.

## Data and processing strategies

### GRACE gravity data (ocean mass change)

We adopt the new Tongji-Grace2018 solution, CSR RL06, GFZ RL06, JPL RL06 and ITSG-Grace2018 to estimate the GMOM changes. The Spherical Harmonic (SH) coefficients of GRACE solutions up to degrees and orders (d/o) 60 are downloaded from the website of the International Centre for Global Earth Models (ICGEM) (http://icgem.gfz-potsdam.de). Note that the Tongji-Grace2018 solution was not directly downloaded from the ICGEM website, but recalculated for extending the period from April 2002 to December 2016. We adopt similar post-processing strategies as Chen et al.^4^, including the P4M6 decorrelation filter^24^, 300-km Gaussian smoothing, 500-km buffer zone for reducing signal leakage from land to ocean, replacing GRACE C_20_ term with that from the NASA Goddard Space Flight Center (GSFC)^25^, correcting GIA effects with ICE6G‐D model^26^, and correcting the geocenter motion effects using the degree-1 products, etc. Three degree-1 products of CSR, GFZ and JPL solutions are taken from GRACE Technical Note 13^27^ (TN-13) and the degree-1 products corresponding to Tongji-Grace2018 and ITSG-Grace2018 solutions are computed using the same method as GRACE TN-13 that was proposed by Sun et al.^28^. Besides, there exists a systematic annual phase lag (~ 10°) between Altimetry–Argo and GRACE estimates due to the total mass of the atmosphere is not conserved^12^. Therefore we removed the global mean atmosphere mass change using the GRACE GAA products to correct the annual phase lag following the method of Chen et al.^12^. Note that only GFZ and JPL provide the GAA products, we use the GAA product from GFZ for the correction of CSR RL06, ITSG-Grace2018 and Tongji-Grace2018 solutions. To be consistent with satellite altimetry and Argo observations, the time series of GMOM change is computed by averaging all grids between latitudes 64.5^o^S ~ 64.5^o^N, with the cosine latitude weighting. The time series of GRACE, altimetry and Argo observations are all fitted with least-squares fitting by introducing the offset, linear trend, annual and semi-annual terms. Note that the periods of 161 days and 3.73 years are removed for correcting the S2 and K2 ocean tide aliasing^29–31^.

### Altimetry data (total sea level change)

Satellite altimetry observations of global SSH are available since 1992 when the TOPEX/Poseidon radar altimeter mission was launched. The merged Mean Sea Level Anomalies (MSLA) from TOPEX/Poseidon, Jason-1/2, ERS-1/2, and Envisat observations are provided by the Archiving, Validation, and Interpretation of Satellite Oceanographic (AVISO) data (called Altimetry^a^) (http://www.aviso.oceanobs.com/). The 0.25° × 0.25° daily altimetry SSH data (Altimetry^b^) are averaged into monthly intervals to compute the total global sea-level change (https://cds.climate.copernicus.eu/), Besides, we further use the 0.25° × 0.25° grid SSH data of the CMEMS (Copernicus Marine Environment Monitoring Service) global ocean ensemble reanalysis products (Altimetry^c^), which are produced with a numerical ocean model constrained with data assimilation of satellite and in situ observations (http://marine.copernicus.eu/). The monthly Global Mean Total Sea Level (GMTSL) changes are computed from the three altimetry SSH anomaly grids over the global ocean between latitudes of 64.5°S and 64.5°N. Normally the GIA impact over oceans is directed corrected by adding a constant value of − 0.30 mm/year^32,33^. Considering a 500-km buffer zone from coastal lines is introduced, the mean GIA impact is recomputed as − 0.28 mm/year using the data downloaded from https://www.atmosp.physics.utoronto.ca/~peltier/data.php in our study. Besides considering that the OBD effect cannot be observed by Altimetry, we compute the OBD effect using GRACE solutions as Vishwakarma et al.^9^ and the details can be referred to Ref.^9,14^.

### Argo data (steric sea level change)

The shallow steric sea-level change (< 2000 m) is determined from three gridded subsurface Argo ${1}^{ \circ } \times 1^{ \circ }$ products (including temperature, salinity and pressure datasets), which are provided by the International Pacific Research Center (IPRC), the Scripps Institute of Oceanography (SIO) and China Second Institute of Oceanography (CSIO) respectively. Steric sea-level change can be computed based on the Argo products as^2^,

3 $$ SL_{{{\text{steric}}}} = - \frac{1}{{\rho_{0} }} \cdot \int_{ - h}^{0} {\Delta \rho \cdot dz} $$

where $\rho_{0}$ is the mean density of seawater (1028 kg/m^3^), and $\Delta \rho$ the density change as a function of temperature (*T*), salinity (*S*) and Pressure (*P*)^34^. Here we compute the average steric sea-level changes over global oceans with the latitudes from 64.5° S to 64.5° N using three Argo products IPRC, SIO and CSIO, except for that from the Japan Agency for Marin-Earth Science and Technology (JAMSTEC) due to its available latitude from 60.5° S to 70.5° N. Same as Vishwakarma et al.^9^ and World Climate Research Programme Sea Level Budget Group^13^, the deep-ocean steric sea-level change is taken as + 0.10 mm/year over the period from Jan. 2005 to Dec. 2016.

## Results and analysis

### Global mean ocean mass change from altimetry and argo

Three altimetry products are adopted to compute the time series of GMTSL change for the period from Jan. 2005 to Dec. 2016. The averaged GMTSL time series of three altimetry products is computed and the GMTSL change rate is 3.75 ± 0.12 mm/year. Then we use three Argo products provided by IPRC, SIO and CSIO to compute the Global Mean Steric Sea Level (GMSSL) change rate between the latitude 64.5° S and 64.5° N, which is 1.19 ± 0.08 mm/year (including deep steric sea-level contribution + 0.10 mm/year). The OBD effects are estimated with five GRACE solutions (same post-processing strategies as “GRACE gravity data (ocean mass)” section), the results are presented in Table 1 and the average linear trend of the OBD effects is − 0.10 ± 0.01 mm/year. Hence, the OBD effect to the global mean sea-level change rate is equal to that of deep-ocean steric sea-level change (> 2000 m) (+ 0.10 mm/year), which results in the same misclosure between Eqs. (1) and (2).

**Table 1:** Amplitudes of annual and semi-annual components and linear trends of OBD derived from five GRACE solutions.

Global mean sea level	Annual amplitude [mm] Phase [deg]	Semiannual amplitude [mm] Phase [deg]	Linear trend [mm/year]
CSR RL06	[0.67 ± 0.02] [98.4 ± 1.8]	[0.08 ± 0.02] [237.1 ± 15.8]	− 0.10 ± 0.02
GFZ RL06	[0.67 ± 0.02] [98.3 ± 1.9]	[0.08 ± 0.02] [231.5 ± 17.7]	− 0.09 ± 0.03
JPL RL06	[0.68 ± 0.02] [98.9 ± 1.8]	[0.09 ± 0.02] [234.3 ± 14.7]	− 0.10 ± 0.02
ITSG-Grace2018	[0.70 ± 0.02] [99.1 ± 1.8]	[0.07 ± 0.02] [229.6 ± 22.7]	− 0.10 ± 0.02
Tongji-Grace2018	[0.69 ± 0.02] [98.7 ± 1.9]	[0.08 ± 0.02] [232.5 ± 18.3]	− 0.11 ± 0.02
Averaged OBD	[0.68 ± 0.01] [98.7 ± 1.8]	[0.08 ± 0.01] [233.1 ± 16.5]	− 0.10 ± 0.01

The uncertainty represents the least-squares fitting error (1 sigma for amplitudes and phases and 2 sigmas for trends).

**Figure 1:**
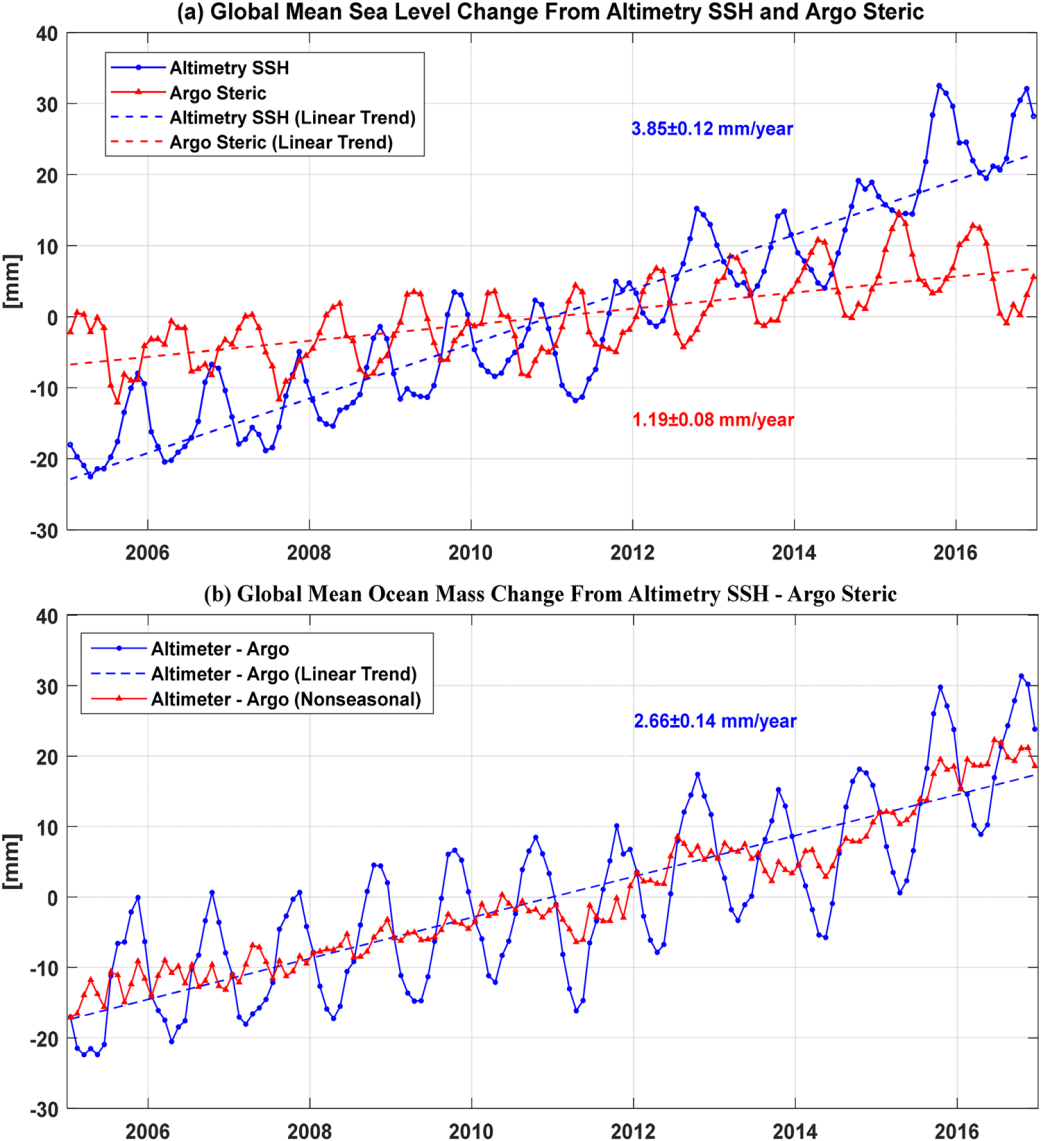
(**a**) GMTSL change derived by satellite altimetry and steric change estimation from the average of three Argo products (IPRC, SIO and CSIO) over the period from January 2005 to December 2016. (**b**) Monthly GMOM change derived from altimetry minus Argo (Altimetry–Argo).

After the linear trend of OBD effects is deducted, the GMTSL change rate is 3.85 ± 0.12 mm/year. The GMTSL and GMSSL time series are shown in Fig. 1a, and the time series of GMOM change derived by GMTSL minus GMSSL is also shown in Fig. 1b with the rate of 2.66 ± 0.14 mm/year. Considering there exist 17 missing months in the GRACE solutions from Jan. 2005 to Dec. 2016, if the same missing months are deleted from the altimetry data, the recomputed GMTSL change rate is 3.76 ± 0.12 mm/year, with a 0.09 mm/year smaller relative to 3.85 ± 0.12 mm/year. However, if the same missing months are deleted from the Argo data, the re-estimated GMSSL change rate will be 1.16 ± 0.08 mm/year, slightly smaller than 1.19 ± 0.08 mm/year. Therefore, the correspondent GMOM change rate derived from Altimetry–Argo is 2.60 ± 0.14 mm/year, with a 0.06 mm/year smaller than 2.66 ± 0.14 mm/year before deleting the GRACE missing months. Note that the amplitudes of annual and semi-annual components and linear trends are estimated by the least-squares fitting approach, the results are presented in Table 2 for the individual and averaged Altimetry and Argo data.

**Table 2:** Amplitudes of annual and semiannual components and linear trends of global mean sea-level change from Altimetry and Argo observations for the period January 2005 to December 2016.

Index	Annual amplitude [mm] Phase [deg]	Semi-annual amplitude [mm] Phase [deg]	Linear trend [mm/year]
**GMTSL**			
Altimetry^a^	[6.17 ± 0.39] [302.4 ± 3.7]	[1.30 ± 0.39] [233.4 ± 18.1]	3.73 ± 0.16
Altimetry^b^	[5.62 ± 0.36] [305.4 ± 3.8]	[1.20 ± 0.36] [248.1 ± 17.1]	3.65 ± 0.14
Altimetry^c^	[5.06 ± 0.48] [308.2 ± 5.9]	[1.73 ± 0.48] [244.8 ± 15.8]	3.87 ± 0.20
Averaged altimetry	[5.62 ± 0.32] [305.1 ± 3.5]	[1.40 ± 0.32] [242.2 ± 15.4]	3.75 ± 0.12
Averaged altimetry*	[5.41 ± 0.32] [305.3 ± 3.5]	[0.93 ± 0.32] [256.6 ± 25.5]	3.66 ± 0.12
Altimetry = averaged altimetry*-OBD	[6.03 ± 0.33] [302.4 ± 4.1]	[0.86 ± 0.33] [258.7 ± 28.8]	3.76 ± 0.12
**GMSSL**			
IPRC	[5.06 ± 0.25] [85.5 ± 2.9]	[0.73 ± 0.25] [226.0 ± 34.0]	1.01 ± 0.10
SIO	[5.35 ± 0.26] [83.1 ± 2.8]	[1.56 ± 0.26] [251.6 ± 9.7]	1.14 ± 0.12
CSIO	[4.60 ± 0.26] [90.7 ± 3.2]	[1.16 ± 0.26] [264.3 ± 12.8]	1.12 ± 0.10
Averaged Argo	[5.00 ± 0.23] [86.2 ± 2.6]	[1.13 ± 0.23] [244.5 ± 9.6]	1.09 ± 0.08
Averaged Argo*	[4.99 ± 0.24] [84.9 ± 2.7]	[1.03 ± 0.24] [247.0 ± 13.3]	1.06 ± 0.08
Argo = averaged Argo* + deep steric (> 2000 m)	[4.99 ± 0.24] [84.9 ± 2.7]	[1.03 ± 0.24] [247.0 ± 13.3]	1.16 ± 0.08
**GMOM**			
Altimetry–Argo	[10.44 ± 0.37] [285.5 ± 2.0]	[0.26 ± 0.37] [24.3 ± 81.6]	2.60 ± 0.14

The uncertainty represents the least-squares fitting error (1 sigma for amplitudes and phases and 2 sigmas for trends). Same missing months are deleted from Altimetry and Argo data, the corresponding re-estimated results are given as (*).

### Global mean ocean mass change from GRACE and altimetry–argo

**Figure 2:**
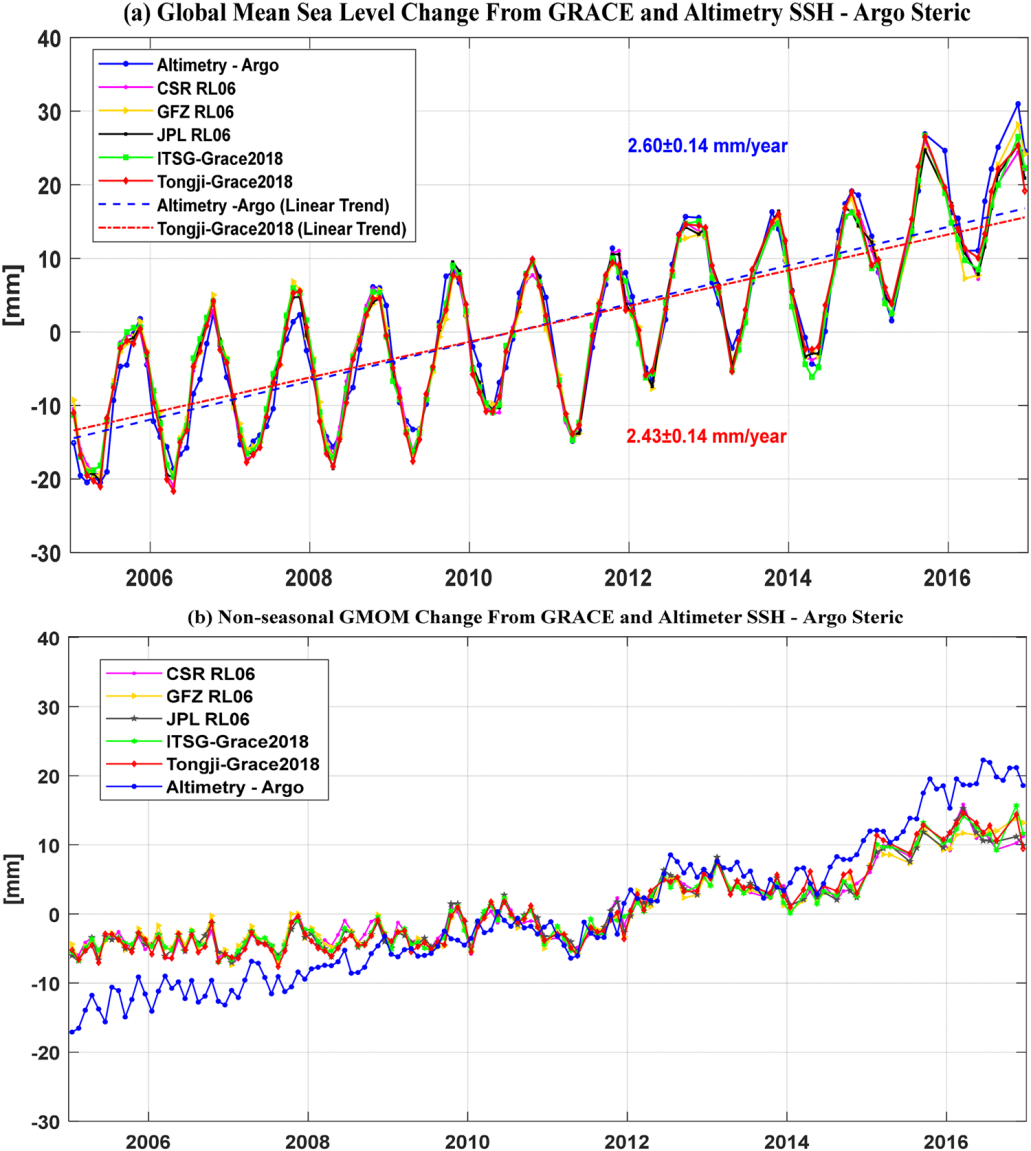
(**a**) GMOM change estimation derived from altimetry minus Argo (Altimetry–Argo) and five GRACE solutions over the period January 2005 and December 2016, (**b**) Nonseasonal monthly GMOM changes derived from GRACE and Altimetry–Argo.

In this section, the GMOM changes are computed from five recent GRACE solutions (CSR RL06, GFZ RL06, JPL RL06, ITSG-Grace2018 and Tongji-Grace2018) to investigate the global sea-level budget over the period from Jan. 2005 to Dec. 2016. The resulted time series of GMOM change from five GRACE solutions and Altimetry–Argo are presented in Fig. 2a and the time series of non-seasonal GMOM changes are presented in Fig. 2b. We can find from Fig. 2 that the linear trend of GMOM change derived from Altimetry–Argo is larger than the GRACE counterparts. The least-squares fitting results of the GMOM changes from Altimetry–Argo and five GRACE solutions are shown in Table 3, where the annual components from GRACE solutions agree well with those from Altimetry–Argo, however, the semi-annual components have a relatively larger difference, especially for the phase components. Moreover, after deleting the GRACE missing months from Altimetry–Argo data the misclosure of the linear trend is reduced by 0.06 mm/year relative to the five GRACE solutions. Compared to the other four GRACE solutions with the GMOM change rates ranging from 2.21 ± 0.14 mm/year to 2.32 ± 0.14 mm/year, Tongji-Grace2018 solution with the change rate of 2.43 ± 0.14 mm/year is the closest to 2.60 ± 0.14 mm/year from Altimetry–Argo, with the misclosure of the global sea level budget reduced about 0.11–0.22 mm/year.

**Table 3:** Amplitudes and phases of annual and semiannual components and linear trends of GMOM change from altimetry–Argo and GRACE observations over the period from January 2005 to December 2016.

Global mean sea level	Annual amplitude [mm] Phase [deg]	Semiannual amplitude [mm] Phase [deg]	Linear trend [mm/year]
Altimetry–Argo	[10.44 ± 0.37] [285.5 ± 2.0]	[0.26 ± 0.37] [24.3 ± 81.6]	2.60 ± 0.14
CSR RL06	[9.72 ± 0.33] [283.4 ± 1.9]	[0.82 ± 0.33] [31.4 ± 23.1]	2.30 ± 0.14
GFZ RL06	[9.69 ± 0.34] [284.0 ± 2.0]	[0.82 ± 0.34] [24.0 ± 23.6]	2.26 ± 0.14
JPL RL06	[9.83 ± 0.33] [283.7 ± 1.9]	[0.92 ± 0.33] [30.8 ± 20.2]	2.32 ± 0.14
ITSG-Grace2018	[10.0 ± 0.34] [282.2 ± 1.9]	[0.77 ± 0.34] [20.8 ± 25.1]	2.21 ± 0.14
Tongji-Grace2018	[9.81 ± 0.34] [282.2 ± 2.0]	[0.87 ± 0.34] [26.7 ± 22.3]	2.43 ± 0.14

**Figure 3:**
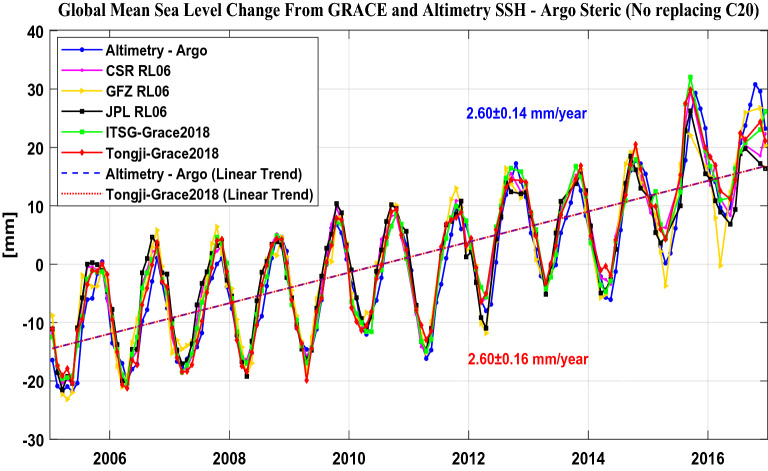
GMOM change estimation derived from altimetry minus Argo (Altimetry–Argo) and five GRACE solutions over the period from January 2005 to December 2016 without replacing the C_20_ term.

Normally the GRACE C_20_ term is replaced with that by satellite laser ranging (SLR)^35^, however, Chen et al.^4^ found that the misclosure of the global sea-level budget derived from CSR RL06 will be reduced when the GRACE C_20_ term is retained. Therefore, we further re-estimate the GMOM changes by retaining the C_20_ terms for all GRACE solutions and present the results in Fig. 3 and Table 4. From Table 4, we can find that the linear trends of all GRACE solutions become larger except for the JPL RL06 solution. The re-estimated linear trends are 2.41 ± 0.14 mm/year for CSR RL06, 2.60 ± 0.16 mm/year for Tongji-Grace2018 and 2.54 ± 0.16 mm/year for ITSG-Grace2018, respectively, all of them are better closed relative to 2.60 ± 0.14 mm/year for Altimetry–Argo after retaining the GRACE C_20_ term, however, just the opposite for JPL RL06 model. In addition, when the GRACE C_20_ term is retained, the uncertainties of the linear trend, annual and semi-annual amplitudes are almost all larger than those in Table 3 except for CSR and JPL RL06 model.

**Table 4:** Amplitudes and phases of annual and semiannual components and linear trends of GMOM change from altimetry–Argo and GRACE data from January 2005 to December 2016 (no replacing C_20_).

GMOM change	Annual amplitude [mm] Phase [deg]	Semiannual amplitude [mm] Phase [deg]	Linear trend [mm/year]
Altimetry–Argo	[10.44 ± 0.37] [285.5 ± 2.0]	[0.26 ± 0.37] [24.3 ± 81.6]	2.60 ± 0.14
CSR RL06	[9.72 ± 0.35] [283.9 ± 2.1]	[0.72 ± 0.35] [44.7 ± 3.0]	2.41 ± 0.14
GFZ RL06	[10.18 ± 0.38] [281.6 ± 2.1]	[1.43 ± 0.38] [24.0 ± 23.6]	2.30 ± 0.16
JPL RL06	[10.01 ± 0.33] [283.9 ± 1.9]	[1.19 ± 0.33] [21.8 ± 15.7]	2.12 ± 0.14
ITSG-Grace2018	[9.85 ± 0.40] [280.8 ± 2.3]	[0.81 ± 0.40] [35.5 ± 27.7]	2.54 ± 0.16
Tongji-Grace2018	[9.80 ± 0.39] [280.6 ± 2.2]	[0.71 ± 0.38] [30.5 ± 30.9]	2.60 ± 0.16

### Contribution of low-degree SH coefficients to GMOM change

To understand why ITSG-Grace2018 and Tongji-Grace2018 solutions have smaller misclosures of global sea-level budget, the contributions of degree-1, C_20_ and other low-degree SH coefficients to the GMOM change are analyzed with the five GRACE solutions.

#### Degree-1 coefficients (C_10_, C_11_ and S_11_)

**Figure 4:**
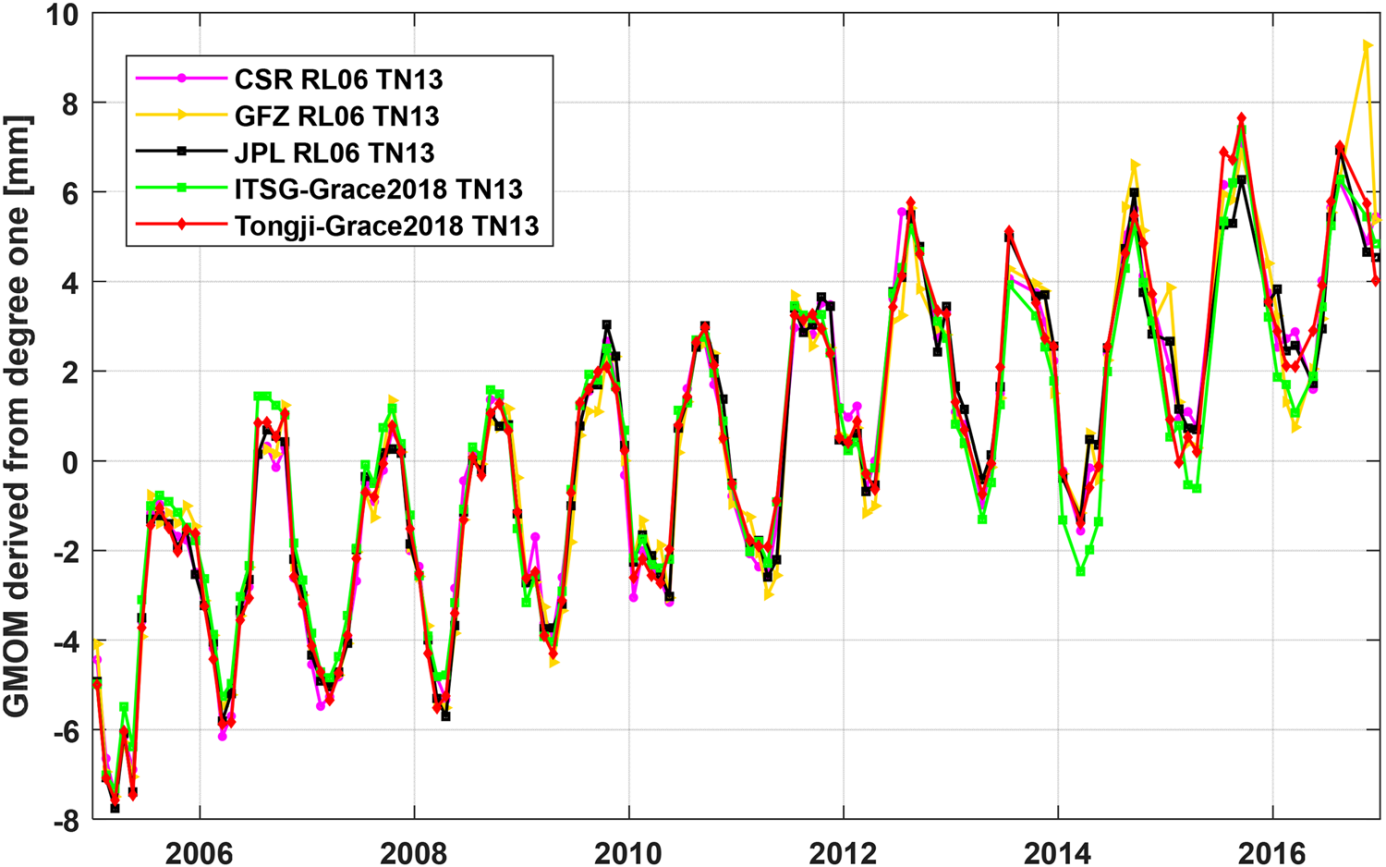
GMOM change estimation derived from degree-1 coefficients over the period January 2005 and December 2016.

The degree-1 coefficients should be added to correct the effects of geocenter motion on the GMOM change^36^. The degree-1 coefficients of CSR RL06, GFZ RL06 and JPL RL06 are provided in GRACE TN-13 products and the degree-1 coefficients corresponding to ITSG-Grace2018 and Tongji-Grace2018 solutions are computed using the same methods as the TN-13 products^27,28^. The GMOM change series derived from the five degree-1 coefficients are shown in Fig. 4 over the period from Jan. 2005 and Dec. 2016. The corresponding statistical results of the GMOM change estimated by the least-squares fitting are presented in Table 5, in which the linear trend of ITSG-Grace2018 solution is 0.60 ± 0.04 mm/year, smaller than that of the other four solutions with about 0.71 ± 0.04 mm/year.

**Table 5:** Amplitudes and phases of annual and semiannual components and linear trends of GMOM change from degree-1 coefficients from January 2005 to December 2016.

Index	Annual amplitude [mm] Phase [deg]	Semiannual amplitude [mm] Phase [deg]	Linear trend [mm/year]
CSR RL06	[2.71 ± 0.11] [255.8 ± 2.2]	[0.43 ± 0.11] [48.3 ± 18.3]	0.72 ± 0.04
GFZ RL06	[2.81 ± 0.11] [261.3 ± 2.3]	[0.38 ± 0.11] [44.3 ± 4.4]	0.71 ± 0.04
JPL RL06	[2.71 ± 0.11] [257.4 ± 2.2]	[0.42 ± 0.11] [52.4 ± 15.1]	0.71 ± 0.04
ITSG-Grace2018	[2.91 ± 0.11] [254.6 ± 2.1]	[0.38 ± 0.11] [46.2 ± 26.2]	0.60 ± 0.04
Tongji-Grace2018	[2.85 ± 0.10] [254.3 ± 2.0]	[0.42 ± 0.10] [44.9 ± 0.6]	0.71 ± 0.04

#### C_20_ coefficients from GRACE and GSFC SLR solutions

**Figure 5:**
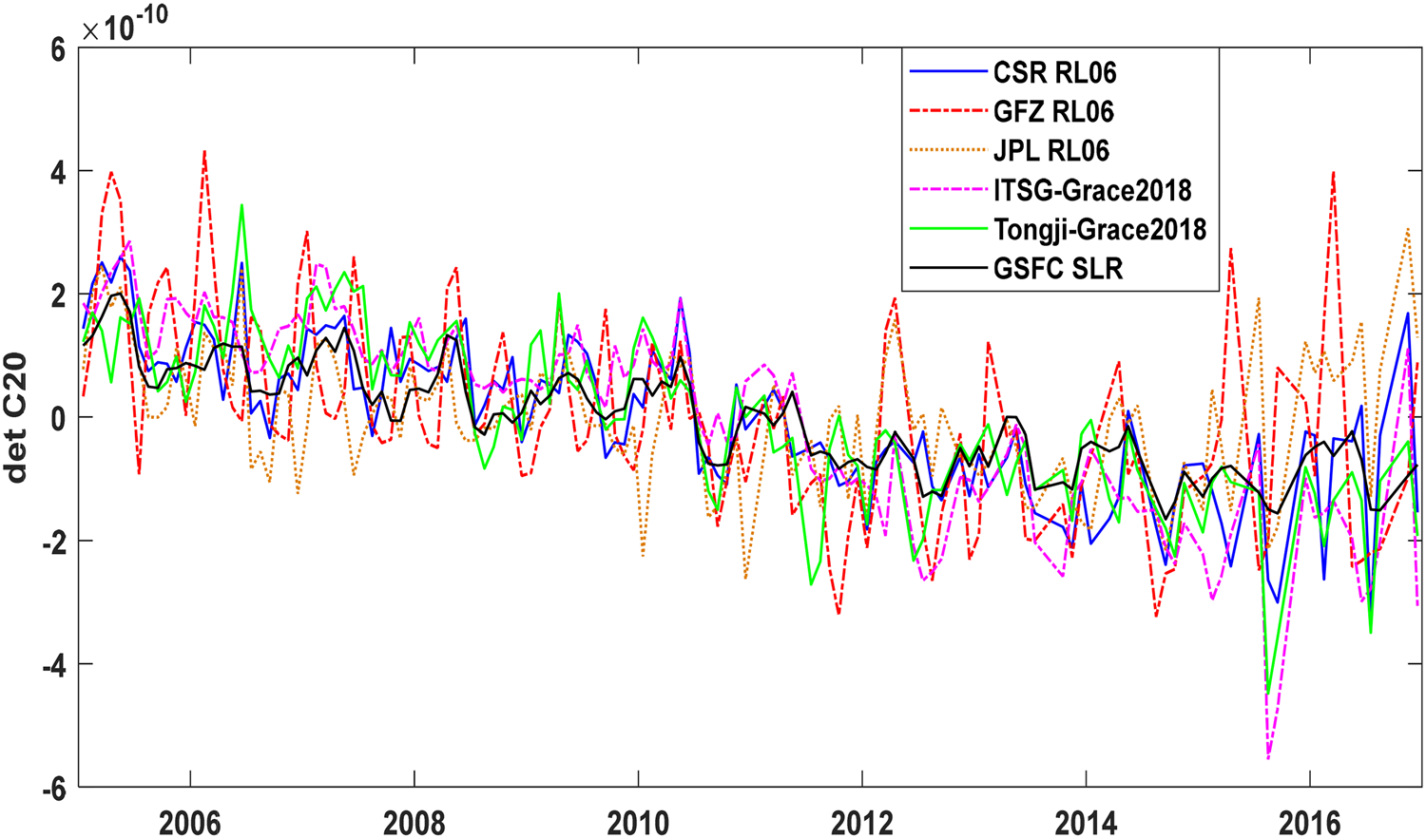
Comparison of $\Delta {\text{C}}_{20}$ from five GRACE solutions and NASA GSFC SLR solution.

Normally the GRACE C_20_ coefficient is replaced by that from the SLR solution due to its relatively large uncertainty. To analyze the contribution of the C_20_ term more clearly, we show the C_20_ coefficients of five GRACE solutions and NASA GSFC SLR solution in Fig. 5 and the annual and semi-annual amplitudes and phases and linear trends of GMOM change derived from the six C_20_ coefficients in Table 6. In Table 6, the linear trend from the C_20_ term of ITSG-Grace2018 solution reaches 0.68 ± 0.06 mm/year, significantly larger than 0.35 ± 0.01 mm/year of NASA GSFC SLR solution, resulting in an obvious increasing trend from 2.21 ± 0.14 mm/year (Table 3) to 2.54 ± 0.16 mm/year (Table 4) after retaining the C_20_ term. Except for the C_20_ term, the low-degree SH coefficients of the ITSG-Grace2018 solution will be further investigated in the next sub-section to show whether it has an obvious difference from the RL06 official solutions and Tongji-Grace2018.

**Table 6:** Amplitudes and phases of annual and semiannual components and linear trends of GMOM change from GRACE and GSFC SLR ΔC_20_ from January 2005 to December 2016.

Index	Annual amplitude [mm]	Semiannual amplitude [mm]	Linear trend [mm/year]
NASA GSFC SLR	0.75 ± 0.06	0.40 ± 0.06	0.35 ± 0.02
CSR RL06	0.73 ± 0.16	0.55 ± 0.15	0.46 ± 0.06
GFZ RL06	1.35 ± 0.26	0.60 ± 0.26	0.40 ± 0.10
JPL RL06	0.92 ± 0.20	0.43 ± 0.20	0.15 ± 0.08
ITSG-Grace2018	0.70 ± 0.16	0.61 ± 0.16	0.68 ± 0.06
Tongji-Grace2018	0.83 ± 0.16	0.44 ± 0.16	0.54 ± 0.06

#### Other low-degree SH coefficients

**Figure 6:**
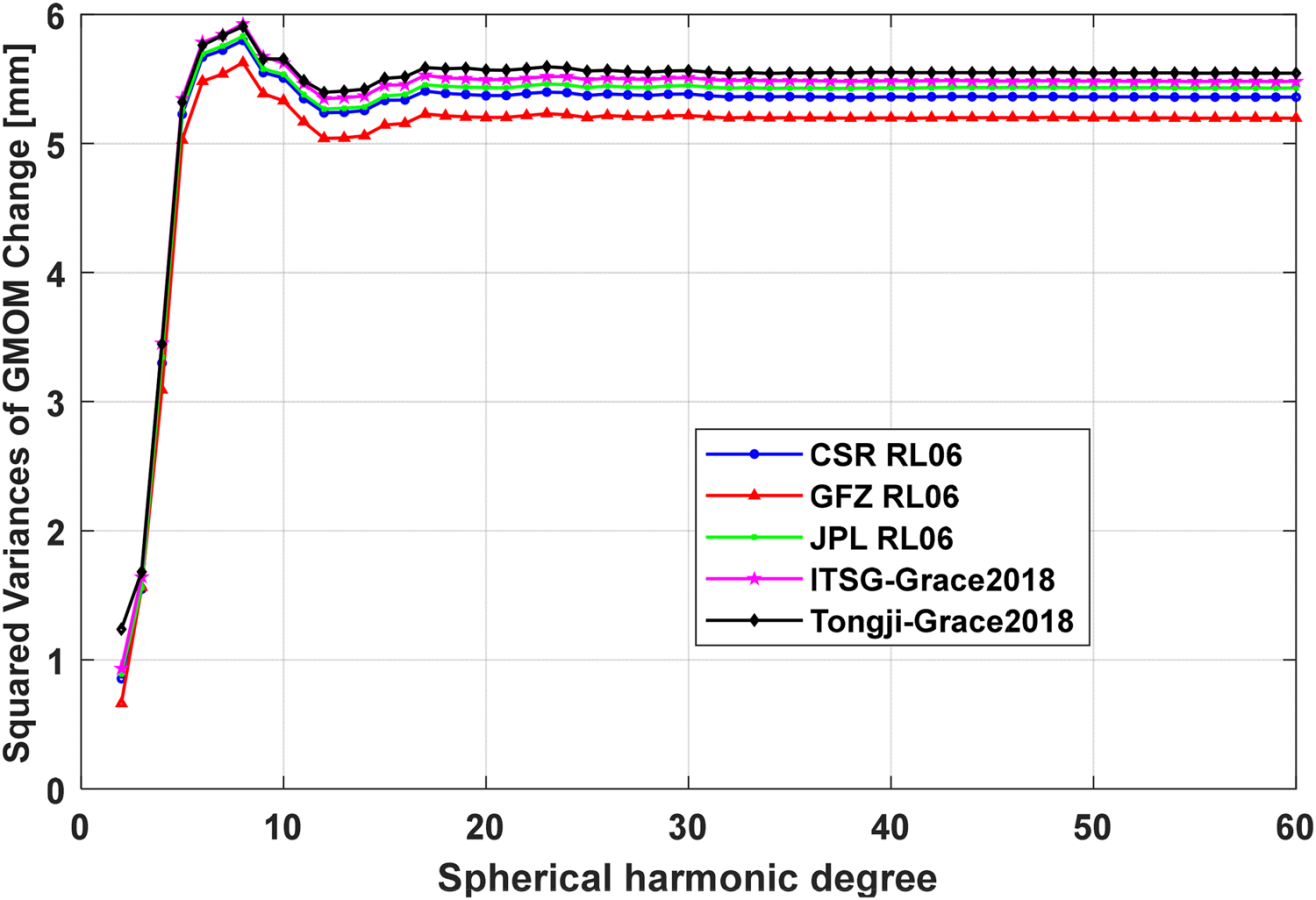
The squared variance of GMOM change series derived from different truncated degrees and orders with five GRACE solutions except for C_20_ term.

To better understand the contributions of other low-degree SH coefficients of five GRACE solutions to GMOM change, the filtered GRACE SH coefficients from 2 to 60 degrees and orders (d/o) except for C_20_ term are transformed to ${1}^{ \circ } \times 1^{ \circ }$ gridded maps of Equivalent Water Height (EWH) respectively. Then the GMOM change time series is computed by averaging all grids over the global oceans between the latitudes 64.5° S–64.5° N excluding ocean grids within 500-km from the coastal lines. The squared variance $\sigma (n)$ of GMOM change derived from the SH coefficients truncated to d/o n is computed to evaluate the ocean signals as,

4 $$ \sigma (n){ = }\sqrt {\sum\limits_{t = 1}^{N} {{\text{GMOM}}_{n}^{2} (t)} /N} $$

where, ${\text{GMOM}}_{n} (t)$ stand for the GMOM change derived from the SH coefficients up to d/o *n*, $N$ is the number of available months. The squared variances of the five GRACE solutions are shown in Fig. 6 for the SH coefficients truncated to different degrees and orders. From Fig. 6, we can find that all squared variances of the Tongji-Grace2018 solution are larger than those of the other four GRACE solutions, indicating that the Tongji-Grace2018 solution can capture more ocean signals. Besides, the squared variances of five solutions all increase when the SH coefficients are truncated from d/o 2 to 8 and then decrease from d/o 9 to 12, which is worthy to be investigated in future and probably caused by the SH representation of gravity solutions. And the squared variances tend to be stable after d/o 20 and approach to 5.37 mm (CSR RL06), 5.20 mm (GFZ RL06), 5.43 mm (JPL RL06), 5.49 mm (ITSG-Grace2018) and 5.57 mm (Tongji-Grace2018), respectively. The statistical results of the squared variances of five GRACE solutions are shown in Table 7 (linear trends), Table 8 (annual amplitude) and Table 9 (semi-annual amplitude). In Table 7, the linear trends truncated to d/o 15 and 20 are very close to those of d/o 60 SH coefficients. Therefore, we can conclude that the Tongji-Grace2018 solution can reduce the misclosure of the global sea-level budget because its low-degree SH coefficients can capture more ocean signals than the other solutions.

**Table 7:** The linear trends of GMOM change for five GRACE solutions up to different degree and orders (d/o) from January 2005 to December 2016 except for the degree-1 and C_20_ coefficients [unit: mm/year].

Index	d/o 10	d/o 15	d/o 20	d/o 60
CSR RL06	0.21 ± 0.12	0.29 ± 0.12	0.34 ± 0.13	0.31 ± 0.13
GFZ RL06	0.16 ± 0.13	0.24 ± 0.12	0.30 ± 0.12	0.27 ± 0.12
JPL RL06	0.22 ± 0.12	0.30 ± 0.12	0.36 ± 0.12	0.34 ± 0.12
ITSG-Grace2018	0.23 ± 0.13	0.31 ± 0.13	0.36 ± 0.13	0.33 ± 0.13
Tongji-Grace2018	0.34 ± 0.13	0.42 ± 0.13	0.47 ± 0.13	0.44 ± 0.13

**Table 8:** The annual amplitude of GMOM change for five GRACE solutions at different truncated degree and orders (d/o) from January 2005 to December 2016 except for the degree-1 and C_20_ coefficients [unit: mm].

Index	d/o 10	d/o 15	d/o 20	d/o 60
CSR RL06	6.90 ± 0.31	6.57 ± 0.31	6.51 ± 0.31	6.57 ± 0.31
GFZ RL06	6.64 ± 0.31	6.31 ± 0.30	6.28 ± 0.31	6.34 ± 0.31
JPL RL06	6.94 ± 0.31	6.62 ± 0.30	6.58 ± 0.31	6.64 ± 0.31
ITSG-Grace2018	7.01 ± 0.32	6.57 ± 0.32	6.51 ± 0.32	6.57 ± 0.32
Tongji-Grace2018	6.90 ± 0.32	6.59 ± 0.32	6.53 ± 0.32	6.58 ± 0.32

**Table 9:** The semi-annual amplitude of GMOM change for five GRACE solutions at different truncated degree and orders (d/o) from January 2005 to December 2016 except for the degree-1 and C_20_ coefficients [unit: mm].

Index	d/o 10	d/o 15	d/o 20	d/o 60
CSR RL06	0.62 ± 0.31	0.63 ± 0.31	0.67 ± 0.31	0.62 ± 0.31
GFZ RL06	0.66 ± 0.31	0.68 ± 0.30	0.73 ± 0.31	0.69 ± 0.31
JPL RL06	0.70 ± 0.31	0.73 ± 0.30	0.79 ± 0.31	0.75 ± 0.31
ITSG-Grace2018	0.65 ± 0.32	0.67 ± 0.32	0.70 ± 0.32	0.66 ± 0.32
Tongji-Grace2018	0.65 ± 0.32	0.67 ± 0.32	0.72 ± 0.32	0.69 ± 0.32

## Conclusions

The new solution Tongji-Grace2018 is used to estimate the GMOM change together with the other four solutions (CSR RL06, GFZ RL06, JPL RL06 and ITSG-Grace 2018), and investigate the misclosure relative to the GMOM change from Altimetry–Argo. The same post-processing strategies as Refs.^4^ and^12^ are adopted for all five GRACE solutions. The results show that all annual amplitudes and phases agree well with those of Altimetry–Argo, the linear trend of the Tongji-Grace2018 solution can reduce the misclosure by 0.11–0.22 mm/year than the other four GRACE solutions since the low-degree SH coefficients of Tongji-Grace2018 solution can capture more ocean signals, which are validated by the statistical results of the squared variances of GMOM series derived from five GRACE solutions with the SH coefficients truncated to different degrees and orders. If the GRACE C_20_ term is retained, the linear trends of the Tongji-Grace2018 and ITSG-Grace2018 solutions are closed with the Altimetry–Argo. If the same month’s data as the GRACE solutions are deleted from the altimetry and Argo data, the misclosure of the sea-level budget will be reduced by 0.06 mm/year. On the whole, the Tongji-Grace2018 solution possesses the smallest misclosure of the global sea-level budget compared to the other four solutions, regardless of that whether GRACE C_20_ is replaced or retained.

## References

[1] Cazenave A, Palanisamy H, Ablain M (2018). Contemporary sea level changes from satellite altimetry: What have we learned? What are the new challenges?. Adv. Space Res..

[2] Chen JL, Wilson CR, Tapley BD, Hu XG (2006). Thermosteric effects on interannual and long-term global mean sea level changes. J. Geodesy.

[3] Church, J. *et al.* Sea level change. In *Climate Change 2013: The Physical Science Basis. Contribution of Working Group I to the Fifth Assessment Report of the Intergovernmental Panel on Climate Change* (eds Stocker, T. *et al.*) 1137–1216. (Cambridge University Press, Cambridge, and New York, NY, 2013).

[4] Chen JL, Tapley BD, Save H, Tamisiea ME, Bettadpur S, Ries J (2018). Quantification of ocean mass change using gravity recovery and climate experiment, satellite altimeter, and Argo floats observations. J. Geophys. Res. Solid Earth.

[5] Chambers DP, Wahr J, Nerem RS (2004). Preliminary observations of global ocean mass variations with GRACE. Geophys. Res. Lett..

[6] Cazenave A (2009). Sea level budget over 2003–2008: a reevaluation from GRACE space gravimetry, satellite altimetry and Argo. Global Planet. Change.

[7] Leuliette EW, Miller L (2009). Closing the sea level rise budget with altimetry, Argo, and GRACE. Geophys. Res. Lett..

[8] Leuliette EW, Willis JK (2011). Balancing the sea level budget. Oceanography.

[9] Vishwakarma BD, Royston S, Riva R, Westaway RM, Bamber JL (2020). Sea level budgets should account for ocean bottom deformation. Geophys. Res. Lett..

[10] Chambers DP (2017). Evaluation of the global mean sea level budget between 1993 and 2014. Surv. Geophys..

[11] Dieng HB, Cazenave A, Meyssignac B, Blain M (2017). New estimate of the current rate of sea level rise from a sea level budget approach. Geophys. Res. Lett..

[12] Chen J, Tapley B, Seo K-W, Wilson C, Ries J (2019). Improved quantification of global mean ocean mass change using GRACE satellite gravimetry measurements. Geophys. Res. Lett..

[13] World Climate Research Programme/WCRP Sea Level Budget Group (2018). Global sea level budget (1993-present). Earth Syst. Sci. Data.

[14] Frederikse T, Riva R, King MA (2017). Ocean bottom deformation due to present-day mass redistribution and its impact on sea level observations. Geophys. Res. Lett..

[15] Dobslaw H (2020). Gravitationally consistent mean barystatic sea level rise from leakage-corrected monthly GRACE data. J. Geophys. Res. Solid Earth.

[16] Dieng HB, Cazenave A, von Schuckmann K, Ablain M, Meyssignac B (2015). Sea level budget over 2005–2013: missing contributions and data errors. Ocean Sci..

[17] Wahr J, Nerem RS, Bettadpur SV (2015). The pole tide and its effect on GRACE time-variable gravity measurements: Implications for estimates of surface mass variations. J. Geophys. Res. Solid Earth.

[18] Jeon T, Seo K-W, Youm K, Chen JL, Wilson CR (2018). Global sea level change signatures observed by GRACE satellite gravimetry. Sci. Rep..

[19] Blazquez A, Meyssignac B, Lemoine JM, Berthier E, Ribes A, Cazenave A (2018). Exploring the uncertainty in grace estimates of the mass redistributions at the earth surface: implications for the global water and sea level budgets. Geophys. J. Int.

[20] Chen J, Tapley B, Wilson C, Cazenave A, Seo K-W, Kim J-S (2020). Global ocean mass change from GRACE and GRACE follow-on and altimeter and Argo measurements. Geophys. Res. Lett..

[21] Uebbing B, Kusche J, Rietbroek R, Landerer FW (2019). Processing choices affect ocean mass estimates from GRACE. J. Geophys. Res. Oceans.

[22] Chen Q, Shen Y, Chen W, Francis O, Zhang X, Chen Q (2019). An optimized short-arc approach: methodology and application to develop refined time series of Tongji-Grace2018 GRACE monthly solutions. J. Geophys. Res-Sol. EA.

[23] Meyer, U. *et al.* Combination service for time-variable gravity fields: operational GRACE-FO combination and validation of Chinese GRACE time-series. In *EGU2021 Conference* (2021).

[24] Chen JL, Wilson CR, Tapley BD, Blankenship DD, Ivins ER (2007). Patagonia ice field melting observed by GRACE. Geophys. Res. Lett..

[25] Loomis BD, Rachlin KE, Luthcke SB (2019). Improved Earth oblateness rate reveals increased ice sheet losses and mass-driven sea level rise. Geophys. Res. Lett..

[26] Peltier RW, Argus DF, Drummond R (2018). Comment on an assessment of the ICE-6G_C (VM5a) glacial isostatic adjustment model” by Purcell. J. Geophys. Res. Solid Earth.

[27] Landerer, F. Monthly estimates of degree-1 (geocenter) gravity coefficients, generated from GRACE (04-2002-06/2017) and GRACE-FO (06/2018 onward) RL06 solutions, GRACE Technical Note 13, The GRACE Project, NASA Jet Propulsion Laboratory. https://podaac-tools.jpl.nasa.gov/drive/files/allData/grace/docs/ (2019).

[28] Sun Y, Riva R, Ditmar P (2016). Optimizing estimates of annual variations and trends in geocenter motion and J2 from a combination of GRACE data and geophysical models. J. Geophys. Res. Solid Earth..

[29] Seo KW, Wilson CR, Han SC, Waliser DE (2008). Gravity recovery and climate experiment (GRACE) alias error from ocean tides. J. Geophys. Res. Solid Earth.

[30] Chen JL, Wilson CR, Seo KW (2009). S2 tide aliasing in grace time-variable gravity solutions. J. Geodesy.

[31] Jeon T, Seo KW, Youm K, Chen J, Wilson CR (2018). Global sea level change signatures observed by GRACE satellite gravimetry. Sci. Rep..

[32] Argus DF, Peltier WR, Drummond R, Moore AW (2014). The Antarctica component of postglacial rebound model ICE-6G_C(VM5a) based on GPS positioning, exposure age dating of ice thicknesses, and relative sea level histories. Geophys. J. Int..

[33] Peltier WR, Argus DF, Drummond R (2015). Space geodesy constrains ice age terminal deglaciation: the global ICE-6GC (VM5a) model. J. Geophys. Res. Solid Earth.

[34] Fofonoff, P., & Millard, R. C., Jr. Algorithms for computation of fundamental properties of seawater, UNESCO Technical Paper in Marine Science vol 44, 53. UNESCO, Paris, pp 17–18. (1983).

[35] Cheng, M. K., & Ries, J. R. Monthly estimates of C20 from 5 SLR satellites based on GRACE RL05 models, GRACE Technical Note 07 Center for Space Research, University of Texas at Austin (2012).

[36] Swenson S, Chambers D, Wahr J (2008). Estimating geocenter variations from a combination of GRACE and ocean model output. J. Geophys. Res..

